# Biodiversity seen through the perspective of insects: 10 simple rules on methodological choices and experimental design for genomic studies

**DOI:** 10.7717/peerj.6727

**Published:** 2019-04-30

**Authors:** Pável Matos-Maraví, Camila Duarte Ritter, Christopher J. Barnes, Martin Nielsen, Urban Olsson, Niklas Wahlberg, Daniel Marquina, Ilari Sääksjärvi, Alexandre Antonelli

**Affiliations:** 1 Department of Biological and Environmental Sciences, University of Gothenburg, Gothenburg, Sweden; 2 Gothenburg Global Biodiversity Centre, Gothenburg, Sweden; 3 Institute of Entomology, Biology Centre CAS, České Budějovice, Czech Republic; 4 Department of Eukaryotic Microbiology, University of Duisburg-Essen, Essen, Germany; 5 Natural History Museum of Denmark, University of Copenhagen, Copenhagen, Denmark; 6 Section for Evolutionary Genomics, Department of Biology, University of Copenhagen, Copenhagen, Denmark; 7 Department of Biology, Lund University, Lund, Sweden; 8 Department of Bioinformatics and Genetics, Swedish Museum of Natural History, Stockholm, Sweden; 9 Department of Zoology, Stockholm University, Stockholm, Sweden; 10 Biodiversity Unit, University of Turku, Turku, Finland; 11 Royal Botanical Garden, Kew, Richmond, Surrey, UK

**Keywords:** Evolution, High-throughput sequencing, Insect genomics, Museomics, Taxonomic impediment

## Abstract

Massively parallel DNA sequencing opens up opportunities for bridging multiple temporal and spatial dimensions in biodiversity research, thanks to its efficiency to recover millions of nucleotide polymorphisms. Here, we identify the current status, discuss the main challenges, and look into future perspectives on biodiversity genomics focusing on insects, which arguably constitute the most diverse and ecologically important group among all animals. We suggest 10 simple rules that provide a succinct step-by-step guide and best-practices to anyone interested in biodiversity research through the study of insect genomics. To this end, we review relevant literature on biodiversity and evolutionary research in the field of entomology. Our compilation is targeted at researchers and students who may not yet be specialists in entomology or molecular biology. We foresee that the genomic revolution and its application to the study of non-model insect lineages will represent a major leap to our understanding of insect diversity.

## Introduction

The global decline in biodiversity is unquestionable (Barnosky et al., 2011). The rate of species diversity loss is comparable to those of ancient mass-extinction events (Ceballos et al., 2015). However, our understanding of the mechanisms that form and maintain species diversity and the impact of environmental disturbances on biodiversity remains limited. Not only do the current methodologies to quantify biodiversity at different temporal and spatial scales need to be profoundly revised (Vellend, 2017), but also a multi-disciplinary effort is necessary to fully understand species diversity and its evolution. In order to maximize efforts when analyzing biodiversity, large datasets need to be generated for hundreds or thousands of specimens with as few steps as possible, following easy-to-implement protocols. Massively parallel DNA sequencing, also called high-throughput sequencing or next-generation sequencing, has been one of the leading technologies for the generation of molecular data since the mid 2000s (Metzker, 2010; Mardis, 2017; Shendure et al., 2017). By using a multiplexing approach, massively parallel sequencing outperforms automated Sanger sequencing in efficiency to recover genomic information, which can be used to understand species diversity variation in time and space.

In this article, we aim to review and to provide a practical guideline on the use of massively parallel DNA sequencing technologies with a focus on one of the largest biotic radiations on Earth: insects. These six-legged invertebrates represent more than half of all known eukaryotic species (Grimaldi & Engel, 2005; Mora et al., 2011; Stork et al., 2015; Stork, 2018) and they are one of the most important components of eukaryotic biodiversity in terms of abundance and ecology. However, as much as 80% of insect diversity, and therefore much of the Earth’s biodiversity, remains to be formally described (Hamilton et al., 2010; Scheffers et al., 2012; Stork, 2018). While there is so much undescribed insect diversity in nature, a significant number may already be deposited within museum collections in need of formal description (Suarez & Tsutsui, 2004; Veijalainen et al., 2012). Therefore, the study of biodiversity through massively parallel sequencing applied to insects, using both mass-sampling techniques in the field and the archived material at public and private collections, is timely and represents a significant opportunity to advance our understanding of life on Earth.

This article fills a gap in the literature in the form of a simple, concise and hopefully easy-to-follow guideline to study biodiversity using insects and massively parallel sequencing. Accordingly, this review is primarily targeted at researchers and students who may not yet be experts in entomology or molecular biology.

### Survey methodology

The authors of this paper are familiar with entomological mass-sampling techniques, specimen preservation and storage for genomic work, massively parallel sequencing and tools for post-sequencing bioinformatics. We discussed the relevant literature on these topics during a two-day workshop titled “Insect diversity and evolution on the era of genomics,” held on the February 27th and 28th, 2017 in Gothenburg, Sweden. During this meeting, we reviewed published literature related to biodiversity and evolutionary research using insects, including but not limited to methods, reviews and original articles. In order to unveil the number of publications using insects and high-throughput sequencing over years, the most popular sequencing platforms and library preparations, we ensured an unbiased procedure by searching the literature stored in the Web of Science^™^ Core Collection on November 22nd, 2018. We used 12 combinations of the keywords: “insect” + “biodiversity”/“museum”/“metabarcoding”/“phylogenom*” + “next generation sequencing”/“high throughput sequencing”/“single molecule sequencing.” We searched for publications from 2006, the year of release of the first truly high-throughput sequencing platform (Goodwin, McPherson & McCombie, 2016), to November 2018. We retrieved a total of 118 publications (File S1) and we filtered this list by type of article (original article, review, others). In addition, based on our expertise, we added to this list 18 relevant original articles that were not retrieved in our search using Web of Science. In total, we selected 91 original articles that generated sequence data by massively parallel sequencing for discussion below (File S2). We acknowledge that this is not a complete list of studies on this topic, but we consider it to be representative for the work being conducted in the last years.

### Ten simple steps to study biodiversity through insect genomics

**Figure 1:**
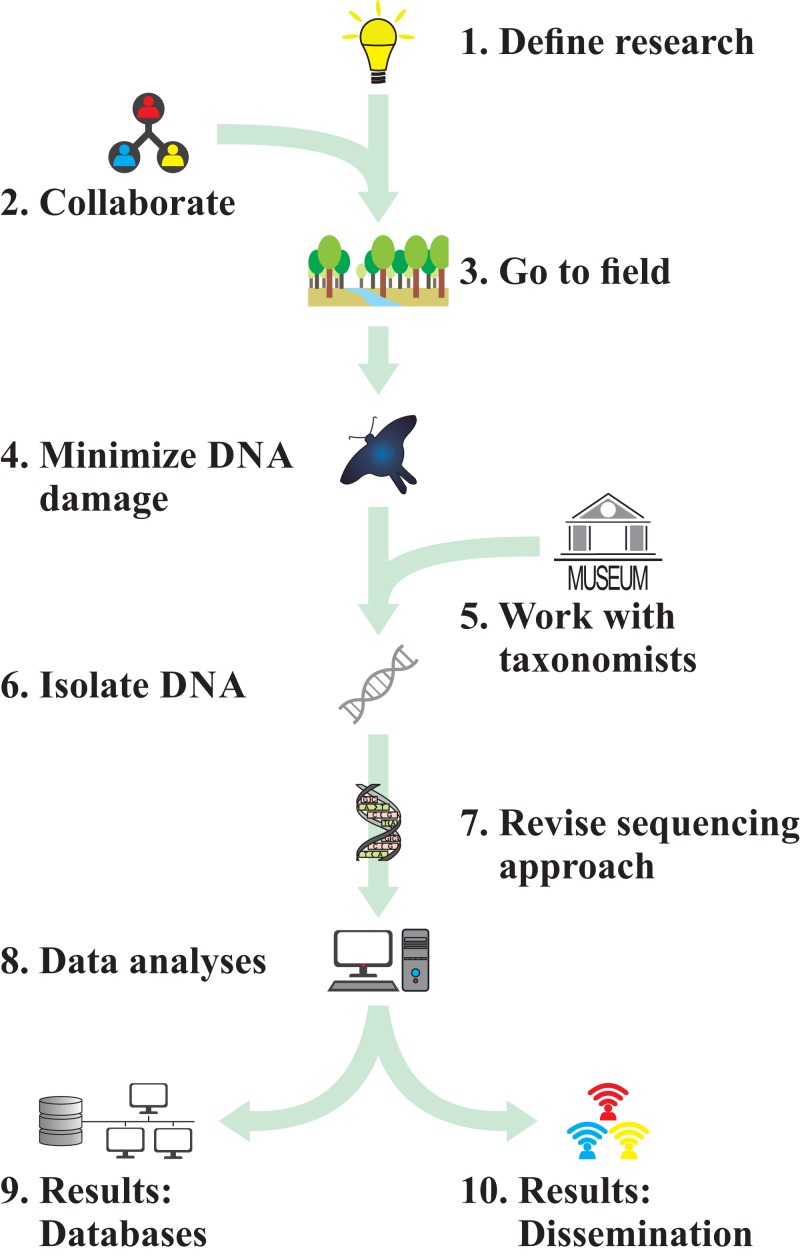
Flowchart illustrating the 10 rules proposed here to study biodiversity through insect genomics.

We structure this article in 10 simple rules (Fig. 1), formulated in a way that we hope will be accessible for readers who may not yet be familiar with entomological or massively parallel sequencing approaches. Based on these recommendations, we hope that readers will eventually be capable of (1) better interpret the results and conclusions coming from published insect biodiversity research, and (2) start planning a multi-dimensional study of biodiversity using insects as target group and high-throughput sequencing. Overall, we briefly review the current state in biodiversity and evolutionary research through the study of insect diversity. We identify a series of limitations and challenges currently faced by these studies, but we also find hopeful approaches to study biodiversity patterns through the perspective of insects.

#### Rule 1: Define the questions and scope of the study

Producing genomic data is no longer a major challenge for many research groups. Instead, many researchers seem to be producing large amounts of data, without always having a clear idea of how to properly use them afterward. Although it may seem obvious, we consider important to stress that careful thinking and planning is required to define the research questions and hypothesis of any study, and how to best address them. This is particularly important when dealing with a data-rich, novel technology such as massively parallel DNA sequencing. A few projects might be totally discovery-driven with no prior expectations, but in general it is preferable to clearly define the hypotheses to be tested *a priori*, and how. This will then inform on the whole chain of methods and analyses. There is no “one size fits all” methodology when it comes to biodiversity and evolutionary studies.

With massively parallel DNA sequencing, the study of evolutionary relations can be complemented with fast quantification of diversity and abundances. It also facilitates research on species interactions such as studies on ecological networks through metabarcoding (Toju, 2015), and in environmental samples (Shokralla et al., 2012) or even from the ethanol used for preservation of historical specimens (Linard et al., 2016). However, economical limitations exist regarding the number of specimens and the extent of their genomes that can be sequenced in a typical project (Wachi, Matsubayashi & Maeto, 2018). Therefore, researchers should choose from a series of available sequencing approaches that best suits their research questions (see Rule 7). For example, if the focus is on finding potential loci involved in adaptation and speciation, a reduced representation of the genomes might be cost-efficient because several individuals from different populations could be pooled in one sequencing experiment. If the aim is instead to profile many organisms within insect communities, DNA metabarcoding may provide a fast quantification of diversity.

#### Rule 2: Set up your collaborations strategically

A major challenge in the study of evolution from populations to species is the lack of non-genomic data, including taxonomic, paleontological, and ecological information. Despite the abundance of genomic information that can nowadays be generated, major challenges remain to (1) increase field expeditions in search of the unknown diversity, (2) incorporate fossil data in phylogenies based on molecular data, and (3) study the phenotypes and life history data in specimen collections. Naturally, the most efficient direction to integrate such different perspectives is to establish and strengthen a collaborative network. For example, working along with paleontologists might bring a temporal perspective in the study of evolution and biodiversity dynamics (Marshall, 2017). Collaborating closely with ecologists would strengthen the study of adaptation and the mechanisms of speciation. A comprehensive knowledge of life history data, insect ecologies, or common garden experiments are ideal to tease apart adaptive from non-adaptive variation. Moreover, natural history museums (NHMs) are the repositories of our natural world and include not only archived specimens but also valuable historical, demographic, life-history, and genetic data that can add additional dimensions to evolutionary research (Burrell, Disotell & Bergey, 2015; Buerki & Baker, 2016). For example, population range expansion in historical times (Ryan et al., 2018), host-parasite interaction changes after human disturbances (Gottdenker et al., 2016), or the effect of current climate change on the structure of populations (Basset et al., 2015), are topics that could be directly benefited by incorporating the information from NHM collection records (Burrell, Disotell & Bergey, 2015).

Collaborative networks are also very important to be more efficient at planning budgets and to set the standards for whole-genome sequencing. For example, the Vertebrate Genomes Project (https://vertebrategenomesproject.org/) is a large collaborative network with the aim to sequence and annotate high-quality genome sequences of all 66,000 extant vertebrate species. Although such large collaborative networks are yet missing for the insect research community, large projects focusing on insect diversity and evolution have been successful at disentangling phylogenetic relationships (e.g., the 1KITE project; http://www.1kite.org/) and for the coordination of efforts for whole genome sequencing among research groups (Sadd et al., 2015).

#### Rule 3: Go to the field

**Figure 2:**
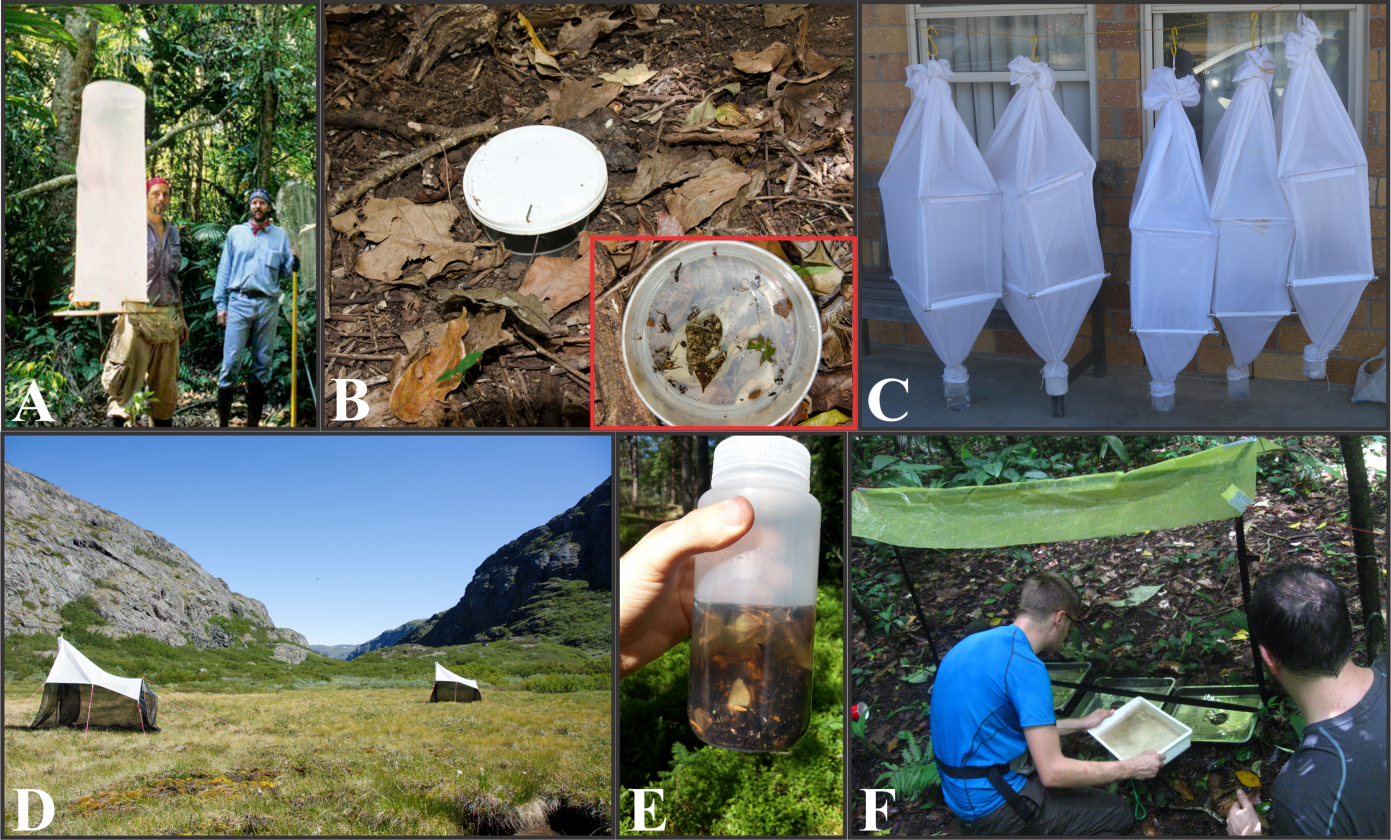
Entomological mass-sampling techniques. (A) Van Someren-Rydon trap, which targets fruit-feeding butterflies. (B) Pitfall trap, which is used to collect forest floor insects—photograph within the red frame depicts the content of pitfall trap. (C) Winkler, an insect collecting device for species inhabiting the leaf litter and soil. (D) Malaise trap, which targets strong-flying insects. (E) The content deposited in the collecting vessel of a Malaise trap. (F) Flight interception, which collects insects flying into the barrier. Photo credits: A, Phil DeVries; B, Martin Nielsen; C, Matthias Seidel; D, Martin Nielsen; E, Daniel Marquina; and F, Emmanuel Arriaga-Varela.

We are worried that the rapid increase of genetic data in public databases might discourage students and researchers from generating novel data. Instead, we argue that field work is absolutely essential to the advancement of our field, and should be part of every biologist’s education as well as part of the routine of more senior researchers. Fieldwork will also benefit museum collections, and vice-versa: museum collections—through genetic and morphological studies based on specimens—will benefit fieldwork. Of course, there might be lines of research that do not demand fieldwork, but even taxonomists, method developers, and researchers in other disciplines may profit from the experience of regularly studying and responsibly collecting specimens or samples in nature. Extensive field surveys are often required to obtain a representative inventory of insect assemblages at both local and regional scales; but such surveys represent only a minority of all entomological field studies. This is problematic given the high species richness and varying abundance, habits and seasonality of insects, including parasitoids, predators, scavengers, leaf-chewers, sap-suckers, among others (Stork, 2018). A careful selection of field sampling methods, along with proper understanding of their function and targeted groups, is thus critical (Noyes, 1989) (see Table 1 for an overview of main mass-sampling methods and Fig. 2).

**Table 1:** Representative description of methods for mass sampling of insects and their application for NGS.

Method	Example	Taxa targeted	Equipment costs	Suitability for genomic research	Sampling effort	Limitations
Trap-sampling	*Van Someren-Rydon*	Fruit-feeding butterflies, from forest floor to canopy	**Low**; negligible if self-built	**Yes**; no killing reagent; baits such as fermenting fruit, faeces, rotting meat	**Minimum**: five traps in forest, 10 traps in open areas; **Collection**: once or twice per day; **Personnel**: two people, collection and record; **Complement**: opportunistic hand collection	Need for long-term data due to different butterfly communities throughout the year; Other feeding guilts are missing, such as nectar-feeding
Trap-sampling	*Pitfall*	Forest floor insects such as dung beetles, flies, ants	**Low**; negligible if self-built	**Depending on killing reagent**; best results if done with detergent and water, propylen glycol; baits such as human dung	**Minimum**: 20 traps per day; linear transect; **Collection**: at least once per day; **Personnel**: one person; **Complement** with flight intercept traps	Lot of ethanol must be replaced every week to prevent DNA decay
Leaf-litter collector	*Mini-Winkler*	Leaf-litter and soil insects, such as ants, beetles	**Medium**	**Yes**; 95% EtOH most commonly used as killing reagent	**Minimum**: 20 collectors, each with one m^2^ leaf litter; **Collection**: once, if extraction is run in parallel; **Personnel**: two people recommended; **Complement** with bait-traps and hand collecting	Limited to forested areas, and not suitable during peak of dry or rain season; No sampling of vegetation-associated, canopy or subterranean insects
Flying-insect collector	*Malaise*	Strong-flying insects, such as Hymenoptera and Diptera	**High**	**Yes**; 95% EtOH most commonly used as killing reagent	**Minimum**: two traps for fast surveys; **Collection**: little care, leave in field for 2–4 weeks; **Personnel**: one person; **Complement** with flight interception traps	Placement of trap in “likely” flight paths, thus a component of subjectivity is introduced
Flying-insect collector	*Flight interception*	Flying insects, such as beetles, cockroaches, crickets	**Low**; negligible if self-built	**Depending on killing reagent**; best results if done in salt-saturated water and detergent, propylen glycol; formaldehyde solutions but in detriment of DNA recovery	**Minimum**: two traps for fast surveys; **Collection**: once or twice per day; **Personnel**: one person; **Complement** with bait and light traps	Ideal for slow-flying insects, which hit the plastic sheet and fall in the container with killing reagent
Insecticidal knockdown	*Canopy fogging*	Arboreal insect community	**High**	**Yes**; insecticide as killing reagent	**Collection**: laborious and problems with pseudoreplication; **Complement** with canopy light trapping and flight interception traps	Canopy access still limited; High demand on logistics; Risk of local environmental damage (minimized through the use of rapidly decaying insecticides)

**Note:**

This is not a comprehensive list and is only aimed at providing an overview of available possibilities of widespread use. In Costs (equipments and consumables per sampling effort), we roughly categorized them as Low (approx. < US $50), Medium (approx. US $50—$100), High (approx. > US $100).

For some cases, such as in biodiversity assessments, it may be enough to conduct simple and rapid field surveys. However, in other cases, such as in exhaustive inventories or when studying diversity dynamics through time and space, greater mass-sampling efforts may be needed. Such campaigns require a combination of multiple methods, longer term inventories and wide expertise, together with effective ways to estimate true species richness based on collected samples (Vogel, 2017). For example, in a recent tropical large-scale species inventory, Borkent & Brown (2015) investigated local species richness of cloud forest Diptera (true flies) for more than 1 year by using two Malaise traps and a wide range of supplementary collecting methods. In addition to these, a 1-week intensive “Diptera-Blitz” was conducted by a large network of experts, inspired on the BioBlitz concept (Lundmark, 2003) which aims at recording most of biodiversity at one locality during a short time period. In another case study, Gómez et al. (2018) sampled the Western Amazonian local parasitoid wasp diversity by using 41 Malaise traps in three separate field campaigns and seasons, with a total sampling effort of 230 Malaise-trap months scattered throughout 1998–2011 (one Malaise-trap month corresponds to one trap collecting in the field for a period of 1 month). In this case, despite the massive sampling effort, cumulative species curves suggested that a significant portion of the local parasitoid diversity remained unobserved; a fact that can be generalized for many other tropical insect groups. Reviews of entomological collection methods for both mass-sampling and group-specific research are available in the literature and are essential reading before field collections (Agosti et al., 2000; Basset et al., 2003; Lamarre et al., 2012; Larsen, 2016).

Needless to say, be a sensible collector! Many insects are rare and threatened, so every collecting effort should be associated with a risk assessment, even informally if not required by law. There are also many federal and international regulations to follow, such as those stipulated under the Nagoya Protocol under the Convention on Biological Diversity (https://www.cbd.int/abs/about/) and the CITES legislation (https://www.cites.org/). In addition, researchers should follow all good practices for Access and Benefit Sharing (e.g., https://naturwissenschaften.ch/organisations/biodiversity/abs/goodpractice), and deposit their specimens in public NHMs.

#### Rule 4: Treat your specimens well to enhance their use

The amount and quality of isolated genomic DNA from insect collections depend on a myriad of factors, including killing reagents, method of preservation of specimens in the field, and final voucher storage conditions (Kanda et al., 2015; Short, Dikow & Moreau, 2018). For example, Dillon, Austin & Bartowsky (1996) (see also Reiss, Schwert & Ashworth, 1995; Gilbert et al., 2007b) found that specimens killed with ethanol yielded significantly higher quantities of high quality DNA compared to other killing/preservation agents such as ethyl acetate vapor, formalin or ethylene glycol. Moreover, rapid and effective drying of the specimens in the field, especially in the tropics, are important for voucher preservation and may be an alternative to freezing-based preservation (Prendini, Hanner & DeSalle, 2002); cryopreservation is the formal name for the technique that uses very low temperatures to preserve tissues and specimens. Initiatives to establish large cryobanks are important (Koebler, 2013), although these technologies are currently limited to very few large and well-funded NHMs (Corthals & Desalle, 2005). Preservation of specimens in ethanol and at low temperatures is ideal, but may cause logistic problems during transportation and would make the collections highly flammable. Propylene glycol may be a safer alternative and logistically easier to transport than ethanol (Ferro & Park, 2013), and it might even be used to attract certain arthropod species (Höfer et al., 2015). The use of ethylene glycol may provide reasonable amounts of DNA regardless of specimen age, and with lesser risks in the field (Dillon, Austin & Bartowsky, 1996).

The age of specimens seems not to be a critical factor for obtaining DNA for massively parallel sequencing (e.g., as in snakes archived in museum collections, (Ruane & Austin, 2017); see also Table 2 for an overview of published studies using archived insects). DNA fragmentation increases with time, while the median fragment sizes decrease, but these changes do not happen linearly over time (Sawyer et al., 2012). Rather than age, preservation and storage methods are in fact better predictors of DNA quality isolated from old specimens (Burrell, Disotell & Bergey, 2015). Evidently, due to the fragmented nature of ancient DNA, PCR-based techniques are overall not successful to recover genetic data. Fortunately, evidence suggests that fragmented DNA due to age or preservation reagents does not dramatically affect the performance of PCR-free, massively parallel sequencing (Tin, Economo & Mikheyev, 2014; Timmermans et al., 2016; Carøe et al., 2018).

**Table 2:** Overview of massively parallel DNA sequencing methods applied to insect museum specimens.

Publication	Taxon group	Samples analyzed	Sequencing approach and platform	Output
Staats et al. (2013)	*Flies and beetles*	**Number**: three specimens; **Age**: 1992–1995; **Tissue**: one to three legs, thorax, whole specimen (destructive protocol)	**Shotgun** whole genome skimming; Illumina HiSeq™ 2000	**Read depth**: 3.5×–146.1× (mt genome); **% Mapping**: 0.002–0.82 (mt genome); **Contamination**: one specimen extensive bacteriophage & fungal DNA
Tin, Economo & Mikheyev (2014)	*Flies and ants*	**Number**: 11 specimens; **Age**: 1910–1976; **Tissue**: whole specimen (non-destructive protocol)	**Shotgun** whole genome skimming; **RAD-tag**; Illumina MiSeq™ & HiSeq™ 2500	**Read depth**: 0.08×–1.0× (whole genome); **% Mapping**: 19–76 (whole genome); **Contamination**: not reported
Heintzman et al. (2014)	*Beetles*	**Number**: four specimens; **Age**: Late Pleistocene (C^14^), 1875–1950 (museum); **Tissue**: one hind leg, pronotum, elytron (destructive protocol)	**Shotgun** whole genome skimming; Illumina HiSeq™ 2000	**Reads aligned** to reference: 0.009%–0.225× (mt genome & five nuclear loci); **% Insect contigs**: 0.25–46.5; **Contamination**: up to ca. 20% mammalian sequences in contigs
Maddison & Cooper (2014)	*Beetles*	**Number**: one specimen; **Age**: 1968; **Tissue**: whole specimen (non-destructive protocol)	**Shotgun** whole genome skimming; Illumina HiSeq™ 2000	**Read depth**: not reported (eight gene targets); **% Gene length** coverage: 95–100 (eight gene targets); **Contamination**: not reported
Kanda et al. (2015)	*Beetles*	**Number**: 13 specimens; **Age**: 1929–2010; **Tissue**: whole specimen (non-destructive protocol)	**Shotgun** whole genome skimming; Illumina HiSeq™ 2000 (two lanes)	**Read depth**: 0.44×–4.64× (67 gene targets); **N50**: 280–700 (67 gene targets); **Contamination**: possible in some specimens but not quantified
Timmermans et al. (2016)	*Butterflies*	**Number**: 35 specimens; **Age**: 1980–2005; **Tissue**: one leg (destructive protocol)	**Shotgun** whole genome skimming; Illumina MiSeq™ (1/3 flow cell)	**% Coverage**: 0–100 (mt coding loci); **Contamination**: not reported; **Failure rate**: four out of 35 specimens any reads matching mt genomes
Suchan et al. (2016)	*Butterflies and grasshoppers*	**Number**: 60 specimens; **Age**: 1908–1997; **Tissue**: legs (destructive protocol)	**Target capture** of RAD probes; Illumina MiSeq™ & HiSeq™ (one lane each)	**Median depth**: 10× (for each SNP); **% Matrix fullness**: 52–72.5 (RAD loci); **Contamination**: ca. 9% of contigs were of exogenous origin
Blaimer et al. (2016)	*Carpenter bees*	**Number**: 51 specimens; **Age**: 1894–2013; **Tissue**: one leg (destructive protocol)	**Target capture** of Hymenopteran UCEs; Illumina MiSeq™	**Average coverage**: 7.4×–182.4× (UCE loci); **Recovered loci**: 6–972 (UCE per sample); **Contamination**: not reported
Pitteloud et al. (2017)	*Butterflies*	**Number**: 32 specimens; **Age**: 1929–2012; **Tissue**: legs (destructive protocol)	**PCR Multiplex & Shotgun sequencing**; Illumina MiSeq™	**Length sequences** (bp): 109–7,297 (mt and rDNA loci); **Contamination**: not reported

**Note:**

This is a selection of studies covering a variety of taxonomic groups, sampling strategies and sequencing approaches.

Despite the advantages of using massively parallel DNA sequencing over Sanger when dealing with old specimens, the success of current sequencing approaches still depends in some cases on the quality of isolated DNA, such as in RAD-seq and single-molecule sequencing. For these reasons, minimal specimen damage in the field and during storage is always strongly advisable.

#### Rule 5: Work closely with taxonomists

The tasks of taxonomists, including the identification, description, and classification of species in meaningful groupings, are unfortunately sometimes neglected. The high diversity and density of insects, coupled with laborious taxonomic assessment and lack of resources for taxonomists, makes the morphological identification of every specimen sampled by mass-collecting techniques a difficult and high resource-consuming task. The so-called “taxonomic impediment” (Di Castri, Vernhes & Younes, 1992) encompasses two general difficulties: (1) not enough resources and training are allocated to taxonomic work and (2) few people are working in taxonomy thus slowing down the rate of species discovery, identification, and classification (Wheeler, Raven & Wilson, 2004; De Carvalho et al., 2007; Ebach, Valdecasas & Wheeler, 2011; Audisio, 2017).

We may be in the midst of a revolution in taxonomy to cope with recent technological advances (Dubois, 2011; Ceríaco et al., 2016; Garnett & Christidis, 2017; Raposo et al., 2017; Thorpe, 2017). In the meantime, entomological research must use complementary approaches to reliably estimate diversity through time and among localities. Therefore, taxonomists should be part of any biodiversity studies using insect genomics, and the DNA sequences generated by such studies should be seen as a necessary supplement to the traditional work of taxonomists.

#### Rule 6: Isolate DNA in the right way

Most recent studies using massively parallel DNA sequencing, even those on ancient insects, have used commercial kits for DNA isolation, thus reducing time, complexity, and health risks in laboratory procedures (Staats et al., 2013; Heintzman et al., 2014; Kanda et al., 2015; Blaimer et al., 2016; Pitteloud et al., 2017). However, in-house methods might be more effective than commercial kits when working with old samples having little and low-quality DNA (e.g., see laboratory protocols in Gilbert et al., 2007c; Meyer et al., 2016). Whenever possible, non-destructive protocols for DNA isolation are preferable when working with valuable, archived specimens or with bulk samples such as those coming from insect mass-collecting techniques that later need to be taxonomically curated. However, there is surprisingly little data available comparing the efficiency of destructive vs. non-destructive protocols applied to insects (but see Gilbert et al., 2007a; Nieman et al., 2015). A number of non-destructive DNA isolation protocols have been proposed (Thomsen et al., 2009; Castalanelli et al., 2010; Tin, Economo & Mikheyev, 2014), but in general they vary depending on the targeted insect group. For example, insects whose external structure are not delicate, including Diptera, Hymenoptera and Coleoptera, tend to be more resistant to submergence of whole specimen in extraction buffers, giving higher DNA yields (Heintzman et al., 2014; Tin, Economo & Mikheyev, 2014). In other more delicate groups such as Lepidoptera, the use of abdomens is advisable, given that in many cases the abdomens need to be removed from the individual for genitalia preparation (Knölke et al., 2005). In other insect groups that hold sufficient starting material for DNA isolation in particular tissues, such as muscles in the massive legs of Orthoptera (grasshoppers, locusts, crickets) and large beetles, grinding one leg might not be a significant loss to the collection (Tagliavia et al., 2011). Inminute insects such asmicrohymenopterans (tiny wasps in the superfamily Chalcidoidea), the use of non-destructive DNA extraction protocols can be complemented with whole genome amplification prior to library preparation for highthroughput sequencing (Cruaud et al., 2018, 2019).

Many curators at NHMs may be reluctant to provide specimens for molecular studies, with valid reasons, since most species might consist of singletons or very rare collections (Lim, Balke & Meier, 2012). The design of selective sampling, minimizing the damage of collections, is therefore crucial. As a side note, there has not been any discussion in the literature about the suitability for massively parallel sequencing using the hundreds of thousands, or perhaps millions, DNA aliquots generated in the past three decades for Sanger-sequencing work. In principle, old DNA aliquots of low quantities and potentially fragmented may face the same constraints of using archived specimens from NHMs or other collections, and might thus be processed using laboratory protocols designed for old specimens (e.g., library preparation, sequencing approach) (Tin, Economo & Mikheyev, 2014; Kanda et al., 2015; Suchan et al., 2016; Timmermans et al., 2016).

Highly-degraded DNA material, such as those coming from museum specimens, might not be suitable for single-molecule DNA sequencing or by certain short-read sequencing protocols such as RADseq (but see protocols that use whole genome amplification prior to reduced-representation sequencing, Cruaud et al., 2018, and targeted sequencing, Cruaud et al., 2019). High molecular weight is only ensured from fresh specimens that have been stored at low temperatures. Moreover, in single-molecule sequencing technologies such as PacBio^®^ (see Rule 7), the required DNA quantity may demand the use of more than one individual when insects are tiny (Pacific Biosciences, 2018). Additionally, dissections of insects prior to genomic DNA isolation might be necessary in single-molecule DNA sequencing, in order to avoid inadvertently sequencing the DNA of symbionts, or when the focus of the study is on a particular insect microbiome (e.g., the gut microbiota).

#### Rule 7: Revise your DNA sequencing approach

At this point, you should already have decided which sequencing approach will be best suitable to address your research question(s), but now you should carefully evaluate the quality of DNA that you de facto were able to obtain, and decide on which sequencing approach to really follow.

**Figure 3:**
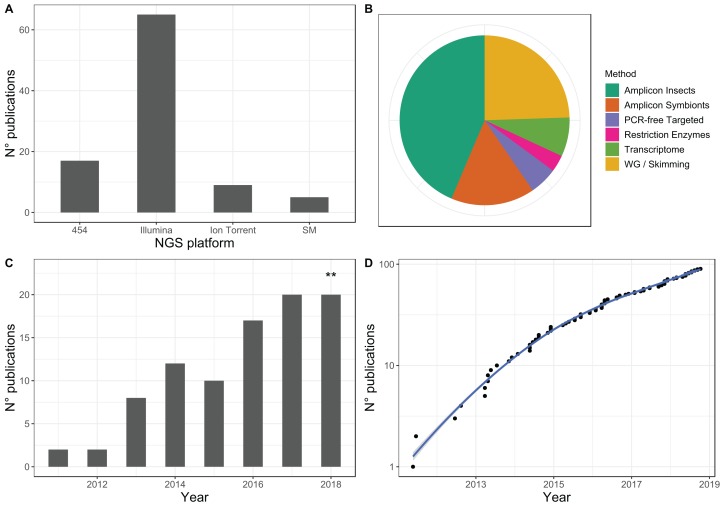
Overview of published studies focusing on insect diversity and evolution using massively parallel sequencing. (A) The main sequencing platforms (SM stands for single-molecule, including those from PacBio and Oxford Nanopore technologies). (B) The main library preparation methods used for high-throughput sequencing (WG stands for whole-genome sequencing). (C) Number of publications by year (**our search was conducted on November 22nd, 2018). (D) Cumulative publications over time (number of publications in logarithmic scale). In general, about 68% of the studies we were able to find (File S2) were conducted in Illumina platform, whereas about 65% of all studies have used some form of targeted sequencing.

Reviews on massively parallel DNA sequencing approaches can be found in the literature (Mamanova et al., 2010; Metzker, 2010; Mardis, 2017). Below, we categorize and briefly describe available massively parallel DNA sequencing technologies of potential interest for entomological biodiversity research (see Table 3 for a summary of such methods and key publications). The current leading short-read DNA sequencing technology is from Illumina, Inc.: approximately 68% of the studies we were able to find that used high-throughput sequencing on insects were conducted using this platform (Fig. 3A). We have grouped the main approaches used in the study of entomological biodiversity into three categories (Table 3): (1) targeted-sequencing, (2) non-targeted, reduced-representation of whole genome, and (3) whole-genome skimming. In addition, emerging single-molecule DNA sequencing technologies, such as those developed by Oxford Nanopore Technologies Ltd. and PacBio (Pacific Biosciences of California, Inc., Menlo Park, CA, USA), can accelerate the amount of DNA data recovery in real time (Thompson & Milos, 2011). We consider these technologies as promising, despite the fact that they have only been recently implemented for the study of insect diversity (e.g., in the genome assembly of a firefly, Coleoptera, (Fu et al., 2017)). Below we provide a summary of these techniques.

**Table 3:** Examples of massively parallel DNA sequencing methods applied to insects.

Approach	Case reference	Main applications	Taxon group	Impact
Whole-transcriptome shotgun	Misof et al. (2014a)	*Phylogenomics*	*Class Insecta*	First phylogenomic study to cover all hexapod orders
Whole-genome shotgun	Tang et al. (2014)	*Mitochondrial metagenomics*	*Several insect orders*	Pioneering proof-of-concept study to show feasibility of PCR-free mitogenome sequence in bulk samples
RAD-seq	Tin, Economo & Mikheyev (2014)	*Phylogenetics; Museomics*	*Flies and ants*	One of the first insect museomic studies using massive parallel sequencing, and a guideline for non-destructive DNA isolation and library preparation
Target capture	Suchan et al. (2016)	*Phylogeography*	*Butterflies and grasshoppers*	New method to target RAD probes (hyRAD). Proof-of-concept using divergent taxa and archived specimens
Target capture	Faircloth et al. (2015)	*Phylogenomics*	*Hymenoptera*	Enrichment of Ultraconserved Elements (UCE) of the Hymenoptera order
Single-molecule	Kelley et al. (2014)	*Comparative genomics*	*Antarctic midge*	Single-molecule real time whole-genome sequencing using PacBio^®^ RS II System

**Note:**

These studies were among the first that used high-throughput methods to investigate insect diversity. A more comprehensive list of published studies is presented in File S2.

**Targeted sequencing:** This is a highly-efficient approach when the aim is to recover DNA markers with a particular rate of evolution (fast and slow) or under different selective pressures (Lemmon & Lemmon, 2013). Moreover, because it targets only a tiny subset of the whole genome, targeted sequencing is cost-effective as tens or hundreds of specimens can be pooled together in a single sequencing experiment (Mamanova et al., 2010). In fact, about 65% of published studies focusing on insects or their symbionts have used some form of targeted sequencing (Fig. 3B). Targeted sequencing is particularly useful when working with environmental samples, such as those coming from mass-sampling techniques (Morinière et al., 2016). For example, metabarcoding, an approach that targets a barcoding region such as the COI mitochondrial gene, can be useful in the study of evolution among environments and in biodiversity assessments. This is because metabarcoding might be more reliable, faster and replicable than traditional biodiversity surveys (Ji et al., 2013; Zhou et al., 2013; Vesterinen et al., 2016), although they should rather be seen as complimentary (Ritter et al., 2019).

There are two usual ways to target particular loci: (1) through PCR or (2) by using “baits”-based in-vitro capture. PCR has the advantage of being cheap but the development of universal primers is the main limitation because sequence specificity to desired loci decreases through mutation and long divergence times among lineages. Nevertheless, PCR-based amplicon sequencing is so far the main method used in published studies with a focus on insects or their symbionts (ca. 60% of reviewed studies; Fig. 3B). On the other hand, target capture using hybridizing baits instead of PCR can be expensive (baits need to be specially synthesized) but has the advantages of (1) simplify laboratory procedures (one can pool several specimens for the capture experiment), (2) target a wider range of lineages despite evolutionary distance among them, (3) reduce amplification biases due to PCR primer design and relative abundance of DNA molecules in a pool of specimens, and (4) it might still work with highly fragmented DNA such as those coming from archived specimens at NHMs.

Prior genomic information, either published annotated genomes or transcriptomes, is needed in order to design target-enrichment probes, which are the hybridizing baits that pull out the targeted loci for sequencing. Probe kits targeting conserved regions primarily for phylogenomic purposes have been published for those insect orders having good genomic reference databases (Faircloth et al., 2015; Faircloth, 2016a; Young et al., 2016; Breinholt et al., 2018). Recent attempts to integrate baits-based capture into metabarcoding have had different degrees of success, such as the sequencing of non-target organisms or pseudogenes on the negative side (Shokralla et al., 2016), or the recovery of sequences of very rare species in a pool of samples and the quantification of relative abundance and biomass on the positive side (Dowle et al., 2016).

**Random reduced-representation of genome:** Restriction-site-associated DNA (RAD) sequencing has proven to be a cost-efficient approach to generate millions of single nucleotide polymorphisms (SNPs), both neutral and under selection (Andrews et al., 2016) RAD-seq is a versatile approach as it has been used in studies on phylogeography (e.g., postglacial range expansions, Emerson et al., 2010), ecology (e.g., habitat association and differentiation of populations, Nice et al., 2019), and evolution (e.g., inference of phylogenetic relationships and genome-wide introgression, Dasmahapatra et al., 2012). However, there are two possible caveats.

Firstly, restriction enzyme sites may not be evolutionarily conserved. Thus, RAD-seq seems to be restricted to populations or closely-related species. However, a recent protocol targeting RAD-seq markers (hyRAD) may ameliorate the lack of phylogenetic conservation of restriction enzyme sites across divergent lineages (Suchan et al., 2016).

Secondly, the amount and quality of DNA might impose a limitation to RAD-seq. For example, Tin, Economo & Mikheyev (2014), using ant specimens as old as 100 years, were able to recover SNPs, but were unsuccessful at genome mapping due to the extremely short DNA fragments and imprecise DNA size selection. Long DNA fragments are needed for an efficient restriction enzyme activity. An alternative reduced-representation method called MIG-seq (Suyama & Matsuki, 2015) might work with moderately fragmented DNA, because it is based on PCR without restriction enzyme digestion steps.

**Whole-genome skimming:** This is the simplest approach in terms of sequence library preparation. It consists of randomly, shallowly sequencing the whole-genome of an individual, including both mitochondrial and nuclear content. Furthermore, when working with historical specimens with highly fragmented DNA, one can skip the step of fragmentation (usually through sonication) during library preparation (Suchan et al., 2016; Timmermans et al., 2016). Whole-genome skimming has been applied in a number of insect studies, proving that the method is fast and can recover entire mitochondrial genomes from even old museum material (Staats et al., 2013), and low-copy nuclear protein-coding genes (Maddison & Cooper, 2014; Kanda et al., 2015).

With the expected decrease in sequencing prices, target sequencing approaches may no longer be a cost-effective choice in the future. For instance, recent studies have identified the benefits of mitochondrial metagenomics (MMG). This technique produces longer barcodes with larger numbers of SNPs, because it uses mitogenomes instead of only the COI fragment, and PCR-free library preparation (Crampton-Platt et al., 2015). This in turn allows the use of highly-fragmented DNA from old specimens, and permits a more reliable quantification of relative abundance (i.e., biomass) in mass-sampling collections (Crampton-Platt et al., 2015, 2016; Cicconardi et al., 2017; Gómez-Rodríguez et al., 2017). However, it has been noted that having a reference genome is important to improve mapping and discovery of homologous SNPs in the nuclear genome (Tin, Economo & Mikheyev, 2014), which may yet restrict the use of whole-genome skimming and the recovery of nuclear data in insect groups with poor genomic information.

**Single-molecule sequencing approaches** such as those developed by PacBio^®^ and Oxford Nanopore Technologies, Ltd. The portability of some devices (e.g., Oxford Nanopore MinION^™^, Oxford, United Kingdom) that can generate DNA sequences in real-time and in virtually any place in the world is a main advantage of these technologies. Indeed, DNA sequencing has already been performed in remote field locations, dealing with for example vertebrates (Menegon et al., 2017) and plants (Parker et al., 2017). The use of MinION^™^ in DNA barcoding in insects has proven to be fast (ca. 2 h), cheap (<USD 2 per sample) and reliable when correction pipelines are used to overcome the still high basecall error rates (>10%) (Mardis, 2017; Shendure et al., 2017; Srivathsan et al., 2018).

Taxonomic biases in bulk material coming from mass-sampling techniques have been reported when working with rDNA amplicons, perhaps associated with the different fragment lengths across insect orders (Krehenwinkel et al., 2018). On the other hand, laboratory protocols are simplified and DNA amplification is not necessary in single-molecule sequencing, which is beneficial for a more accurate quantification of DNA molecules present in the sample pool (Thompson & Milos, 2011). Single-molecule sequencing also promises to drastically reduce costs, meaning that the time when having complete genome sequences for any living insect might be even closer than previously thought (Kelley et al., 2014). Finally, the long reads that single-molecule sequencing approaches generate might help resolve long repeat elements in the genome, thus providing invaluable scaffold for short reads to improve accuracy in assembly and annotation of insect genomes (see Richards & Murali, 2015).

The quality of reference genomes and chromosome-scale scaffolds can be improved by combining long-range and short-read sequencing technologies. For example, PacBio and Nanopore sequencing can overcome repetitive elements by sequencing long DNA fragments, while more accurate short-read sequencing technologies like Illumina can sort out the high error rate of long-range sequencing platforms. For instance, this approach has led to 200-fold increases in contig assembly length and the filling of many gaps in genomes left by short-read approaches only (for example, in avian genome assemblies, Korlach et al., 2017).

#### Rule 8: Choose the most suitable tools for data analyses

Although genomic sequencing is becoming easier and more affordable, processing the data generated remains a major bottleneck in many projects. Bioinformatic pipelines have been implemented during the past two decades of massively parallel sequencing, thus researchers nowadays count with standard procedures to analyze genomic DNA. For example, packages for cleaning and assembling reads exist for bait-based targeted sequencing, such as PHYLUCE (Faircloth, 2016b) and SECAPR (Andermann et al., 2018), as well as for RADseq analysis, such as iPyRAD (Eaton, 2014; Eaton & Overcast, 2016) and Stacks (Rochette & Catchen, 2017). However, there remain limitations and challenges. For example, missing data in supermatrices for phylogenomic studies might hinder statistical power in the inference of species relationships, but their effects in systematic biases are yet unclear (Misof et al., 2014a, 2014b). Moreover, taxonomic sampling in phylogenomics is usually lower than in published Sanger-sequencing work, which may bias systematic inference in insect higher-level phylogenies (Behura, 2015). In general, phylogenomic dataset sizes increase as sequencing costs per base pair decreases over time (Bravo et al., 2018).

A number of pipelines have been published for analyzing amplicon-based, target-sequencing data from environmental samples (Schloss et al., 2009; Caporaso et al., 2010; Boyer et al., 2016). Such programs provide a delimitation of operational taxonomic units (OTUs), the analogs of species, derived from sequence similarity of typically 97%. However, assigning thresholds to define analogs of species is problematic because (1) there is a risk to artificially increase or decrease local diversity as compared to morphology-based taxonomic assessments, (2) inflated OTU richness might be related to sequence chimeras and sequencing errors (but see recent methods to alleviate this; Frøslev et al., 2017), and (3) there is a lack of standardization of threshold values in the literature, reducing the comparability potential of results across studies (Huse et al., 2010; Oliver et al., 2015; Alberdi et al., 2018). The shortcomings of using thresholds to define OTUs might even escalate when studying the entomofauna of hyperdiverse regions such as the tropics. In those cases, there are usually no good estimates of genetic variability between species and a large portion of tropical insects are not represented in reference databases. In any case, the preservation and morphological study of vouchers are critical to validate taxonomic assignments and thresholds.

Mitochondrial metagenomics could in principle improve OTU assignments and species delimitation because contigs span different barcode regions (COI, ND2, 16S rDNA) (Tedersoo et al., 2015; Liu et al., 2016; Srivathsan et al., 2016) and risks of primer-related biases are ameliorated (Taberlet et al., 2012; Tang et al., 2014). Whilst approaches such as log-binomial normalizations (through DeSeq2 and CSS) have attempted to normalize metabarcoding data (McMurdie & Holmes, 2014), results via PCR-based approaches remain semi-quantitative at best (Pawluczyk et al., 2015). However, metagenomic studies of insects have generally been limited only to their microbiomes (Cox-Foster et al., 2007; Suen et al., 2010; Shi et al., 2013). It is difficult to assess the convenience of metagenomics in more complex environmental insect samples because (1) de novo assembly of mixed mitogenomes remains challenging due to the scarcity of reference mitogenomes, and (2) as the number of individuals in a pool increases, sequencing depth needs to be significantly increased in order to get large enough k-mers/contigs to partition different mitogenomes (but see some exceptions in Crampton-Platt et al., 2015, 2016; Cicconardi et al., 2017; Gómez-Rodríguez et al., 2017).

#### Rule 9: Make your data and results publicly available

From a practical viewpoint, what is not in a database does not exist (or nearly so). Databases are not only the repositories of genomic information, but also an indispensable tool in the study of biodiversity and evolution. They also allow the reproduction of results and use for other purposes such as in biodiversity assessments. Biodiversity and evolutionary studies might benefit from the hundreds of insect genome projects already published and registered in GenBank (Yeates et al., 2016) and InsectBase (Yin et al., 2016). In the study of species interactions, such as in host-parasite and feeding habits, a reference database is important because in many cases the identification of taxa through morphological comparison becomes impossible. Examples include the study of internal parasites (Schoonvaere et al., 2016), gut microbiota (Hammer et al., 2017), and highly-degraded organic material as in dietary content (Pompanon et al., 2012).

Initiatives such as BOLD (Ratnasingham & Hebert, 2007) and the widespread usage of the COI barcode will certainly contribute to the assignments of OTU thresholds when studying tropical communities (García-Robledo et al., 2013). However, building local databases that include several markers would complement metabarcoding studies in the identification and delimitation of species (Deagle et al., 2014). Several national reference databases have been implemented or are underway, such as the newly initiated DNAmark project in Denmark (https://www.dnamark.ku.dk/). That initiative aims to provide a reference database for 1,000 species with full mitochondrial sequences, along with nuclear sequences derived from shotgun sequencing. Other initiatives to catalogue national biodiversity have also been put forward in Germany (Hendrich et al., 2015), Norway (NorBOL; http://www.norbol.org/) and Finland (FinBOL; http://www.finbol.org/), which together are further expanding the BOLD project worldwide.

#### Rule 10: Disseminate your findings

Research articles are the standard way to communicate results to the scientific community. However, misinterpretations of scientific findings can be common in the literature aimed for the general public and decision-makers. Thus, public outreach should be explicitly considered as part of project design. Moreover, because scientific research is a collaborative enterprise (see Rule 2), it is important to discuss and reach a consensus with collaborators before spreading findings to the general public. This is particularly important given the recent misunderstandings on biodiversity research that have been reported, and the urge to include both factual evidence and ethical arguments in communications to the general public (Antonelli & Perrigo, 2018).

Given that diversity estimates can fluctuate significantly depending on the way data are produced and analyzed (e.g., as in metabarcoding; Frøslev et al., 2017; Alberdi et al., 2018) special care should be taken when presenting these findings. In general, we advocate for approaches that do not artificially inflate diversity estimates. Furthermore, the access of scientific knowledge and data by governmental bodies is still restricted, especially in low and lower-middle income countries. Biodiversity is a cornerstone in Environmental Impact Assessments, but animal groups such as insects remain underrepresented in biodiversity assessments in species-rich countries (Ritter et al., 2017).

## Perspectives and Conclusions

In this article we have identified general challenges, including: (1) *insufficient evaluation of non-destructive methods applied to insects*, in order to generate DNA of high quantity and quality from fresh, mass-collections and archived specimens, (2) *limitations to genomic data analyses*, including missing genomic information from datasets and methods for estimating diversity and abundance in environmental samples, and (3) *limited taxonomic, ecological, and life history knowledge*, which is not being produced at the same pace as genomic data.

Insects are ideal study organisms because they show remarkable diversity in species number and ecology, being the dominant eukaryotic group in most terrestrial and freshwater environments. The integration of ecology and evolution is achievable with the new massively parallel sequencing approaches, which offer the possibility to generate datasets that can be used in the study of biodiversity at different spatiotemporal scales. For example, the evolutionary framework of local insect communities can now be inferred in a single sequencing effort (Crampton-Platt et al., 2015), while the study of populations and speciation using massively parallel sequencing can be better understood with a comprehensive knowledge of local variations (Jiggins, 2016). Altogether, we expect that the increase of molecular data together with more taxonomic and ecological studies will allow a better understanding of biodiversity and evolution.

## Supplemental Information

10.7717/peerj.6727/supp-1 Supplemental Information 1 Raw data retrieved from our search in Web of Science. Our Literature Review was based on an unbiased search in Web of Science. More details can be found in the main text.

10.7717/peerj.6727/supp-2 Supplemental Information 2 Input data for generating Figure 3. Data was retrieved from Web of Science. The analyses were performed only on Original research. Other type of articles retrieved by our search in Web of Science can be found in the raw data file in Supporting Material.

## Additional Information and Declarations

### Data Availability

The following information was supplied regarding data availability: This article conducted a literature review and retrieved data from Web of Science. The raw data and input data used for the analyses are provided as Supplemental Files.

## References

[ref-1] Agosti D, Majer JD, Alonso LE, Schultz TR (2000). Ants. Standard methods for measuring and monitoring biodiversity.

[ref-2] Alberdi A, Aizpurua O, Gilbert MTP, Bohmann K (2018). Scrutinizing key steps for reliable metabarcoding of environmental samples. Methods in Ecology and Evolution.

[ref-3] Andermann T, Cano Á, Zizka A, Bacon C, Antonelli A (2018). SECAPR—a bioinformatics pipeline for the rapid and user-friendly processing of Illumina sequences, from raw reads to alignments. PeerJ.

[ref-4] Andrews KR, Good JM, Miller MR, Luikart G, Hohenlohe PA (2016). Harnessing the power of RADseq for ecological and evolutionary genomics. Nature Reviews Genetics.

[ref-5] Antonelli A, Perrigo A (2018). The science and ethics of extinction. Nature Ecology & Evolution.

[ref-6] Audisio P (2017). Insect taxonomy, biodiversity research and the new taxonomic impediments. Fragmenta Entomologica.

[ref-7] Barnosky AD, Matzke N, Tomiya S, Wogan GOU, Swartz B, Quental TB, Marshall C, McGuire JL, Lindsey EL, Maguire KC, Mersey B, Ferrer EA (2011). Has the Earth’s sixth mass extinction already arrived?. Nature.

[ref-8] Basset Y, Barrios H, Segar S, Srygley RB, Aiello A, Warren AD, Delgado F, Coronado J, Lezcano J, ARizala S, Rivera M, Perez F, Bobadilla R, Lopez Y, Ramirez JA (2015). The butterflies of Barro Colorado Island, Panama: local extinction since the 1930s. PLOS ONE.

[ref-9] Basset Y, Novotny V, Miller SE, Kitching RL, Basset Y, Novotny V, Miller SE, Kitching RL (2003). Methodological advances and limitations in canopy entomology. Arthropods of Tropical Forests: Spatio-Temporal Dynamics and Resource Use in the Canopy.

[ref-10] Behura SK (2015). Insect phylogenomics. Insect Molecular Biology.

[ref-11] Blaimer BB, Lloyd MW, Guillory WX, Brady SG (2016). Sequence capture and phylogenetic utility of genomic ultraconserved elements obtained from pinned insect specimens. PLOS ONE.

[ref-12] Borkent A, Brown BV (2015). How to inventory tropical flies (Diptera)—one of the megadiverse orders of insects. Zootaxa.

[ref-13] Boyer F, Mercier C, Bonin A, Le Bras Y, Taberlet P, Coissac E (2016). OBITOOLS: a UNIX-inspired software package for DNA metabarcoding. Molecular Ecology Resources.

[ref-14] Bravo GA, Antonelli A, Bacon CD, Bartoszek K, Huynh S, Jones G, Knowles LL, Lamichhaney S, Marcussen T, Nakhleh LK, Oxelman B, Pfeil B, Schliep A, Werneck FP, Wiedenhoeft J, Willows-Munro S, Edwards SV (2018). Embracing heterogeneity: building the tree of life and the future of phylogenomics. PeerJ Preprints.

[ref-15] Breinholt JW, Earl C, Lemmon AR, Lemmon EM, Xiao L, Kawahara AY (2018). Resolving relationships among the megadiverse butterflies and moths with a novel pipeline for anchored phylogenomics. Systematic Biology.

[ref-16] Buerki S, Baker WJ (2016). Collections-based research in the genomic era. Biological Journal of the Linnean Society.

[ref-17] Burrell AS, Disotell TR, Bergey CM (2015). The use of museum specimens with high-throughput DNA sequencers. Journal of Human Evolution.

[ref-18] Caporaso JG, Kuczynski J, Stombaugh J, Bittinger K, Bushman FD, Costello EK, Fierer N, Peña AG, Goodrich JK, Gordon JI, Huttley GA, Kelley ST, Knights D, Koenig JE, Ley RE, Lozupone CA, Mcdonald D, Muegge BD, Pirrung M, Reeder J, Sevinsky JR, Turnbaugh PJ, Walters WA, Widmann J, Yatsunenko T, Zaneveld J, Knight R (2010). QIIME allows analysis of high-throughput community sequencing data. Nature Methods.

[ref-19] Carøe C, Gopalakrishnan S, Vinner L, Mak SST, Sinding M-HS, Samaniego JA, Wales N, Sicheritz-Pontén T, Gilbert MTP (2018). Single-tube library preparation for degraded DNA. Methods in Ecology and Evolution.

[ref-20] Castalanelli MA, Severtson DL, Brumley CJ, Szito A, Foottit RG, Grimm M, Munyard K, Groth DM (2010). A rapid non-destructive DNA extraction method for insects and other arthropods. Journal of Asia-Pacific Entomology.

[ref-21] Ceballos G, Ehrlich PR, Barnosky AD, García A, Pringle RM, Palmer TM (2015). Accelerated modern human–induced species losses: entering the sixth mass extinction. Sciences Advances.

[ref-22] Ceríaco LMP, Gutiérrez EE, Dubois A, Abdala CS, Alqarni AS, Adler K, Adriano EA, Aescht E, Agarwal I, Agatha S, Agosti D, Aguiar AJC, Aguiar JJM, Ahrens D, Aleixo A, Alves MJ, Do Amaral FR, Ananjeva N, Andrade MC, De Andrade MB, Andreone F, Aquino PPU, Araujo PB, Arnaud H, Arroyave J, Arthofer W, Artois TJ, Astúa D, Azevedo C, Bagley JC, Baldo D, Barber-James HM, Bärmann EV, Bastos-Silveira C, Bates MF, Bauer AM, Bauer F, Benine RC, Bennett DJ, Bentlage B, Berning B, Bharti D, Biondo C, Birindelli J, Blick T, Boano G, Bockmann FA, Bogdanowicz W, Böhme W, Borgo E, Borkin L, Bornschein MR, Bour R, Branch WR, Brasileiro CA, Braun JK, Bravo GA, Brendonck L, Brito GRR, Britto MR, Buckup PA, Burckhardt D, Burkhardt U, Busack SD, Campos LA, Canard A, Cancello EM, Caramaschi U, Carpenter JM, Carr M, Carrenho R, Cartaxana A, Carvajal MA, Carvalho GS, De Carvalho MR, Chaabane A, Chagas C, Chakrabarty P, Chandra K, Chatzimanolis S, Chordas SW, Christoff AU, Cianferoni F, Claramunt S, Cogãlniceanu D, Collette BB, Colli GR, Colston TJ, Conradie W, Constant J, Constantino R, Cook JA, Cordeiro D, Correia AM, Cotterill FPD, Coyner B, Cozzuol MA, Cracraft J, Crottini A, Cuccodoro G, Curcio FF, D’Udekem D’Acoz C, D’Elía G, D’Haese CA, Das I, Datovo A, Datta-Roy A, David P, Day JG, Daza JD, De Bisthoven LJ, De La Riva De La Viña IJ, De Muizon C, De Pinna M, Piacentini VDQ, De Sá RO, De Vivo M, Decher J, Dekoninck W, Delabie JHC, Delfino M, Delmastro GB, Delsinne T, Denys C, Denzer W, Desutter-Grandcolas L, Deuti K, De Resbecq TD, Di Dario F, Dinets V, DoNascimiento C, Donoso DA, Doria G, Drewes RC, Drouet E, Duarte M, Durette-Desset MC, Dusoulier F, Dutta SK, Engel MS, Epstein M, Escalona M, Esselstyn JA, Eto K, Faivovich J, Falaschi RL, Falin ZH, Faundez EI, Feijó A, Feitosa RM, Fernandes DS, Fikáček M, Fisher BL, FitzPatrick MJ, Forero D, Franz I, Freitag H, Frétey T, Fritz U, Gallut C, Gao S, Garbino GST, Garcete-Barrett BR, García-Prieto L, García FJ, Garcia PCA, Gardner AL, Gardner SL, Garrouste R, Geiger MF, Giarla TC, Giri V, Glaubrecht M, Glotzhober RC, Godoi FSP, Gofas S, Gonçalves PR, Gong J, Gonzalez VH, González-Orej JA, González-Santillán E, González-Soriano E, Goodman SM, Grandcolas P, Grande L, Greenbaum E, Gregorin R, Grillitsch H, Grismer LL, Grootaert P, Grosjean S, Guarino FM, Guayasamin JM, Guénard B, Guevara L, Guidoti M, Gupta D, Gvoždík V, Haddad CFB, Hallermann J (2016). Photography-based taxonomy is inadequate, unnecessary, and potentially harmful for biological sciences. Zootaxa.

[ref-23] Cicconardi F, Borges PAV, Strasberg D, Oromí P, López H, Pérez-Delgado AJ, Casquet J, Caujapé-Castells J, Fernández-Palacios JM, Thébaud C, Emerson BC (2017). MtDNA metagenomics reveals large-scale invasion of belowground arthropod communities by introduced species. Molecular Ecology.

[ref-24] Corthals A, Desalle R (2005). An application of tissue and DNA banking for genomics and conservation: the Ambrose Monell Cryo-Collection (AMCC). Systematic Biology.

[ref-25] Cox-Foster DL, Conlan S, Holmes EC, Palacios G, Evans JD, Moran NA, Quan P-L, Briese T, Hornig M, Geiser DM, Martinson V, Kalkstein AL, Drysdale A, Hui J, Zhai J, Cui L, Hutchison SK, Simons JF, Egholm M, Pettis JS, Lipkin WI (2007). A metagenomic survey of microbes in honey bee colony collapse disorder. Science.

[ref-26] Crampton-Platt A, Timmermans MJTN, Gimmel ML, Kutty SN, Cockerill TD, Khen CV, Vogler AP (2015). Soup to tree: the phylogeny of beetles inferred by mitochondrial metagenomics of a Bornean rainforest sample. Molecular Biology and Evolution.

[ref-27] Crampton-Platt A, Yu DW, Zhou X, Vogler AP (2016). Mitochondrial metagenomics: letting the genes out of the bottle. GigaScience.

[ref-141] Cruaud A, Groussier G, Genson G, Sauné L, Polaszek A, Rasplus JY (2018). Pushing the limits of whole genome amplification: successful sequencing of RADseq library from a single microhymenopteran (Chalcidoidea, Trichogramma). PeerJ.

[ref-142] Cruaud A, Nidelet S, Arnal P, Weber A, Fusu L, Gumovsky A, Rasplus JY (2019). Optimised DNA extraction and library preparation for minute arthropods: application to target enrichment in chalcid wasps used for biocontrol. BioRxiv.

[ref-147] Dasmahapatra KK, Walters JR, Briscoe AD, Davey JW, Whibley A, Nadeau NJ, Zimin A V, Hughes DST, Ferguson LC, Martin SH, Salazar C, Lewis JJ, Adler S, Ahn S-J, Baker DA, Baxter SW, Chamberlain NL, Chauhan R, Counterman BA, Dalmay T, Gilbert LE, Gordon K, Heckel DG, Hines HM, Hoff KJ, Holland PWH, Jacquin-Joly E, Jiggins FM, Jones RT, Kapan DD, Kersey P, Lamas G, Lawson D, Mapleson D, Maroja LS, Martin A, Moxon S, Palmer WJ, Papa R, Papanicolaou A, Pauchet Y, Ray DA, Rosser N, Salzberg SL, Supple MA, Surridge A, Tenger-Trolander A, Vogel H, Wilkinson PA, Wilson D, Yorke JA, Yuan F, Balmuth AL, Eland C, Gharbi K, Thomson M, Gibbs RA, Han Y, Jayaseelan JC, Kovar C, Mathew T, Muzny DM, Ongeri F, Pu L-L, Qu J, Thornton RL, Worley KC, Wu Y-Q, Linares M, Blaxter ML, Ffrench-Constant RH, Joron M, Kronforst MR, Mullen SP, Reed RD, Scherer SE, Richards S, Mallet J, Owen McMillan W, Jiggins CD (2012). Butterfly genome reveals promiscuous exchange of mimicry adaptations among species. Nature.

[ref-28] De Carvalho MR, Bockmann FA, Amorim DS, Brandao CRF, de Vivo M, de Figueiredo JL, Britski HA, de Pinna MCC, Menezes NA, Marques FPL, Papavero N, Cancello EM, Crisci JV, McEachran JD, Schelly RC, Lundberg JG, Gill AC, Britz R, Wheeler QD, Stiassny MLJ, Parenti LR, Page LM, Wheeler WC, Faivovich J, Vari RP, Grande L, Humphries CJ, DeSalle R, Ebach MC, Nelson GJ (2007). Taxonomic impediment or impediment to taxonomy? A commentary on systematics and the cybertaxonomic-automation paradigm. Evolutionary Biology.

[ref-29] Deagle BE, Jarman SN, Coissac E, Pompanon F, Taberlet P (2014). DNA metabarcoding and the cytochrome c oxidase subunit I marker: not a perfect match. Biology Letters.

[ref-30] Di Castri F, Vernhes JR, Younes T (1992). The network approach for understanding global biodiversity. Biology International. The News Magazine of the International Union of Biological Sciences.

[ref-31] Dillon N, Austin AD, Bartowsky E (1996). Comparison of preservation techniques for DNA extraction from hymenopterous insects. Insect Molecular Biology.

[ref-32] Dowle EJ, Pochon X, Banks JC, Shearer K, Wood SA (2016). Targeted gene enrichment and high-throughput sequencing for environmental biomonitoring: a case study using freshwater macroinvertebrates. Molecular Ecology Resources.

[ref-33] Dubois A (2011). The *International Code of Zoological Nomenclature* must be drastically improved before it is too late. Bionomina.

[ref-143] Eaton DA (2014). PyRAD: assembly of de novo RADseq loci for phylogenetic analyses. Bioinformatics.

[ref-144] Eaton DAR, Overcast I (2016). ipyrad: interactive assembly and analysis of RADseq data sets.

[ref-34] Ebach MC, Valdecasas AG, Wheeler QD (2011). Impediments to taxonomy and users of taxonomy: accessibility and impact evaluation. Cladistics.

[ref-145] Emerson KJ, Merz CR, Catchen JM, Hohenlohe PA, Cresko WA, Bradshaw WE, Holzapfel CM (2010). Resolving postglacial phylogeography using high-throughput sequencing. Proceedings of the National Academy of Sciences of the United States of America.

[ref-35] Faircloth BC (2016a). Identifying conserved genomic elements and designing universal probe sets to enrich them. biorxiv preprint.

[ref-36] Faircloth BC (2016b). PHYLUCE is a software package for the analysis of conserved genomic loci. Bioinformatics.

[ref-37] Faircloth BC, Branstetter MG, White ND, Brady SG (2015). Target enrichment of ultraconserved elements from arthropods provides a genomic perspective on relationships among Hymenoptera. Molecular Ecology Resources.

[ref-38] Ferro ML, Park J-S (2013). Effect of propylene glycol concentration on mid-term DNA preservation of Coleoptera. Coleopterists Bulletin.

[ref-39] Frøslev TG, Kjøller R, Bruun HH, Ejrnæs R, Brunbjerg AK, Pietroni C, Hansen AJ (2017). Algorithm for post-clustering curation of DNA amplicon data yields reliable biodiversity estimates. Nature Communications.

[ref-40] Fu X, Li J, Tian Y, Quan W, Zhang S, Liu Q, Liang F, Zhu X, Zhang L, Wag D, Hu J (2017). Long-read sequence assembly of the firefly *Pyrocoelia pectoralis* genome. GigaScience.

[ref-41] García-Robledo C, Erickson DL, Staines CL, Erwin TL, Kress WJ (2013). Tropical plant-herbivore networks: reconstructing species interactions using DNA barcodes. PLOS ONE.

[ref-42] Garnett ST, Christidis L (2017). Taxonomy anarchy hampers conservation. Nature.

[ref-43] Gilbert MTP, Moore W, Melchior L, Worebey M (2007a). DNA extraction from dry museum beetles without conferring external morphological damage. PLOS ONE.

[ref-44] Gilbert MTP, Sanchez JJ, Haselkorn T, Jewell LD, Lucas SB, Van Marck E, Børsting C, Morling N, Worobey M (2007b). Multiplex PCR with minisequencing as an effective high-throughput SNP typing method for formalin-fixed tissue. Electrophoresis.

[ref-45] Gilbert MTP, Tomsho LP, Rendulic S, Packard M, Drautz DI, Sher A, Tikhonov A, Dalén L, Kuznetsova T, Kosintsev P, Campos PF, Higham T, Collins MJ, Wilson AS, Shidlovskiy F, Buigues B, Ericson PGP, Germonpré M, Götherström A, Iacumin P, Nikolaev V, Nowak-Kemp M, Willerslev E, Knight JR, Irzyk GP, Perbost CS, Fredrikson KM, Harkins TT, Sheridan S, Miller W, Schuster SC (2007c). Whole-genome shotgun sequencing of mitochondria from ancient hair shafts. Science.

[ref-46] Gómez IC, Sääksjärvi IE, Mayhew PJ, Pollet M, Rey del Castillo C, Nieves-Aldrey JL, Broad GR, Roininen H, Tuomisto H (2018). Variation in the species richness of parasitoid wasps (Ichneumonidae: Pimplinae and Rhyssinae) across sites on different continents. Insect Conservation and Diversity.

[ref-47] Gómez-Rodríguez C, Timmermans MJTN, Crampton-Platt A, Vogler AP (2017). Intraspecific genetic variation in complex assemblages from mitochondrial metagenomics: comparison with DNA barcodes. Methods in Ecology and Evolution.

[ref-48] Goodwin S, McPherson JD, McCombie WR (2016). Coming of age: ten years of next-generation sequencing technologies. Nature Reviews Genetics.

[ref-49] Gottdenker NL, Chaves LF, Calzada JE, Peterson JK, Santamaría A, Pineda V, Saldaña A (2016). *Trypanosoma cruzi* and *Trypanosoma rangeli* co-infection patterns in insect vectors vary across habitat types in a fragmented forest landscape. Parasitology Open.

[ref-50] Grimaldi D, Engel MS (2005). Evolution of the insects.

[ref-51] Hamilton AJ, Basset Y, Benke KK, Grimbacher PS, Miller SE, Novotný V, Samuelson GA, Stork NE, Weiblen GD, Yen JDL (2010). Quantifying uncertainty in estimation of tropical arthropod species richness. American Naturalist.

[ref-52] Hammer TJ, Janzen DH, Hallwachs W, Jaffe SP, Fierer N (2017). Caterpillars lack a resident gut microbiome. Proceedings of the National Academy of Sciences of the United States of America.

[ref-53] Heintzman PD, Elias SA, Moore K, Paszkiewicz K, Barnes I (2014). Characterizing DNA preservation in degraded specimens of *Amara alpina* (Carabidae: Coleoptera). Molecular Ecology Resources.

[ref-54] Hendrich L, Morinière J, Haszprunar G, Hebert PDN, Hausmann A, Köhler F, Balke M (2015). A comprehensive DNA barcode database for Central European beetles with a focus on Germany: adding more than 3500 identified species to BOLD. Molecular Ecology Resources.

[ref-55] Höfer H, Astrin J, Holstein J, Spelda J, Meyer F, Zarte N (2015). Propylene glycol—a useful capture preservative for spiders for DNA barcoding. Arachnologische Mitteilungen.

[ref-56] Huse SM, Welch DM, Morrison HG, Sogin ML (2010). Ironing out the wrinkles in the rare biosphere through improved OTU clustering. Environmental Microbiology.

[ref-57] Ji Y, Ashton L, Pedley SM, Edwards DP, Tang Y, Nakamura A, Kitching R, Dolman PM, Woodcock P, Edwards FA, Larsen TH, Hsu WW, Benedick S, Hamer KC, Wilcove DS, Bruce C, Wang X, Levi T, Lott M, Emerson BC, Yu DW (2013). Reliable, verifiable and efficient monitoring of biodiversity via metabarcoding. Ecology Letters.

[ref-58] Jiggins CD (2016). The ecology and evolution of heliconius butterflies.

[ref-59] Kanda K, Pflug JM, Sproul JS, Dasenko MA, Maddison DR (2015). Successful recovery of nuclear protein-coding genes from small insects in museums using Illumina sequencing. PLOS ONE.

[ref-60] Kelley JL, Peyton JT, Fiston-Lavier AS, Teets NM, Yee MC, Johnston JS, Bustamante CD, Lee RE, Denlinger DL (2014). Compact genome of the Antarctic midge is likely an adaptation to an extreme environment. Nature Communications.

[ref-61] Knölke S, Erlacher S, Hausmann A, Miller MA, Segerer AH (2005). A procedure for combined genitalia dissection and DNA extraction in Lepidoptera. Insect Systematics & Evolution.

[ref-62] Koebler J (2013). Earth’s life-forms collected to aid in genetic research.

[ref-63] Korlach J, Gedman G, Kingan SB, Chin C-S, Howard JT, Audet J-N, Cantin L, Jarvis ED (2017). De novo PacBio long-read and phased avian genome assemblies correct and add to reference genes generated with intermediate and short reads. GigaScience.

[ref-64] Krehenwinkel H, Pomerantz A, Henderson JB, Kennedy SR, Lim JY, Swamy V, Shoobridge JD, Patel NH, Gillespie RG, Prost S (2018). Nanopore sequencing of long ribosomal DNA amplicons enables portable and simple biodiversity assessments with high phylogenetic resolution across broad taxonomic scale. biorxiv preprint.

[ref-65] Lamarre GPA, Molto Q, Fine PVA, Baraloto C (2012). A comparison of two common flight interception traps to survey tropical arthropods. ZooKeys.

[ref-66] Larsen TH (2016). Core standardized methods for rapid biological field assessment.

[ref-67] Lemmon EM, Lemmon AR (2013). High-throughput genomic data in systematics and phylogenetics. Annual Review of Ecology, Evolution, and Systematics.

[ref-68] Lim GS, Balke M, Meier R (2012). Determining species boundaries in a world full of rarity: singletons, species delimitation methods. Systematic Biology.

[ref-69] Linard B, Arribas P, Andújar C, Crampton-Platt A, Vogler AP (2016). Lessons from genome skimming of arthropod-preserving ethanol. Molecular Ecology Resources.

[ref-70] Liu S, Wang X, Xie L, Tan M, Li Z, Su X, Zhang H, Misof B, Kjer KM, Tang M, Niehuis O, Jiang H, Zhou X (2016). Mitochondrial capture enriches mito-DNA 100 fold, enabling PCR-free mitogenomics biodiversity analysis. Molecular Ecology Resources.

[ref-71] Lundmark C (2003). BioBlitz: getting into backyard biodiversity. BioScience.

[ref-72] Maddison DR, Cooper KW (2014). Species delimitation in the ground beetle subgenus *Liocosmius* (Coleoptera: Carabidae: *Bembidion*), including standard and next-generation sequencing of museum specimens. Zoological Journal of the Linnean Society.

[ref-73] Mamanova L, Coffey AJ, Scott CE, Kozarewa I, Turner EH, Kumar A, Howard E, Shendure J, Turner DJ (2010). Target-enrichment strategies for next-generation sequencing. Nature Methods.

[ref-74] Mardis ER (2017). DNA sequencing technologies: 2006–2016. Nature Protocols.

[ref-75] Marshall CR (2017). Five palaeobiological laws needed to understand the evolution of the living biota. Nature Ecology & Evolution.

[ref-76] McMurdie PJ, Holmes S (2014). Waste not, want not: why rarefying microbiome data is inadmissible. PLOS Computational Biology.

[ref-77] Menegon M, Cantaloni C, Rodriguez-Prieto A, Centomo C, Abdelfattah A, Rossato M, Bernardi M, Xumerle L, Loader S, Delledonne M (2017). On site DNA barcoding by nanopore sequencing. PLOS ONE.

[ref-78] Metzker ML (2010). Sequencing technologies—the next generation. Nature Reviews Genetics.

[ref-79] Meyer M, Arsuaga JL, De Filippo C, Nagel S, Aximu-Petri A, Nickel B, Martínez I, Gracia A, De Castro JMB, Carbonell E, Viola B, Kelso J, Prüfer K, Pääbo S (2016). Nuclear DNA sequences from the Middle Pleistocene Sima de los Huesos hominins. Nature.

[ref-80] Misof B, Liu S, Meusemann K, Peters RS, Donath A, Mayer C, Frandsen PB, Ware J, Flouri T, Beutel RG, Niehuis O, Petersen M, Izquierdo-Carrasco F, Wappler T, Rust J, Aberer AJ, Aspöck U, Aspöck H, Bartel D, Blanke A, Berger S, Böhm A, Buckley TR, Calcott B, Chen J, Friedrich F, Fukui M, Fujita M, Greve C, Grobe P, Gu S, Huang Y, Jermiin LS, Kawahara AY, Krogmann L, Kubiak M, Lanfear R, Letsch H, Li Y, Li Z, Li J, Lu H, Machida R, Mashimo Y, Kapli P, McKenna DD, Meng G, Nakagaki Y, Navarrete-Heredia JL, Ott M, Ou Y, Pass G, Podsiadlowski L, Pohl H, Von Reumont BM, Schütte K, Sekiya K, Shimizu S, Slipinski A, Stamatakis A, Song W, Su X, Szucsich NU, Tan M, Tan X, Tang M, Tang J, Timelthaler G, Tomizuka S, Trautwein M, Tong X, Uchifume T, Walzl MG, Wiegmann BM, Wilbrandt J, Wipfler B, Wong TKF, Wu Q, Wu G, Xie Y, Yang S, Yang Q, Yeates DK, Yoshizawa K, Zhang Q, Zhang R, Zhang W, Zhang Y, Zhao J, Zhou C, Zhou L, Ziesmann T, Zou S, Li Y, Xu X, Zhang Y, Yang H, Wang J, Wang J, Kjer KM, Zhou X (2014a). Phylogenomics resolves the timing and pattern of insect evolution. Science.

[ref-81] Misof B, Meusemann K, von Reumont BM, Kück P, Prohaska SJ, Stadler PF (2014b). A priori assessment of data quality in molecular phylogenetics. Algorithms for Molecular Biology.

[ref-82] Mora C, Tittensor DP, Adl S, Simpson AGB, Worm B (2011). How many species are there on earth and in the ocean?. PLOS Biology.

[ref-83] Morinière J, Cancian de Araujo B, Lam AW, Hausmann A, Balke M, Schmidt S, Hendrich L, Doczkal D, Fartmann B, Arvidsson S, Haszprunar G (2016). Species identification in Malaise trap samples by DNA barcoding based on NGS technologies and a scoring matrix. PLOS ONE.

[ref-146] Nice CC, Fordyce JA, Bell KL, Forister ML, Gompert Z, DeVries PJ (2019). Vertical differentiation in tropical forest butterflies: a novel mechanism generating insect diversity?. Biology Letters.

[ref-84] Nieman CC, Yamasaki Y, Collier TC, Lee Y (2015). A DNA extraction protocol for improved DNA yield from individual mosquitoes. F1000Research.

[ref-85] Noyes JS (1989). A study of five methods of sampling Hymenoptera (Insecta) in a tropical rainforest, with special reference to the Parasitica. Journal of Natural History.

[ref-86] Oliver AK, Brown SP, Callaham MA, Jumpponen A (2015). Polymerase matters: non-proofreading enzymes inflate fungal community richness estimates by up to 15%. Fungal Ecology.

[ref-87] Pacific Biosciences (2018). Preparing samples for PacBio^®^ whole genome sequencing for de novo Assembly: collection and storage.

[ref-88] Parker J, Helmstetter AJ, Devey Di, Wilkinson T, Papadopulos AST (2017). Field-based species identification of closely-related plants using real-time nanopore sequencing. Scientific Reports.

[ref-89] Pawluczyk M, Weiss J, Links MG, Egaña Aranguren M, Wilkinson MD, Egea-Cortines M (2015). Quantitative evaluation of bias in PCR amplification and next-generation sequencing derived from metabarcoding samples. Analytical and Bioanalytical Chemistry.

[ref-90] Pitteloud C, Arrigo N, Suchan T, Mastretta-Yanes A, Vila R, Dinca V, Hernandez-Roldan J, Brockmann E, Chittaro Y, Kleckova I, Fumagalli L, Buerki S, Alvarez N (2017). Climatic niche evolution is faster in sympatric than allopatric lineages of the butterfly genus Pyrgus. Proceedings of the Royal Society B: Biological Sciences.

[ref-91] Pompanon F, Deagle BE, Symondson WOC, Brown DS, Jarman SN, Taberlet P (2012). Who is eating what: diet assessment using next generation sequencing. Molecular Ecology.

[ref-92] Prendini L, Hanner R, DeSalle R, DeSalle R, Giribet G, Wheeler W (2002). Obtaining, storing and archiving specimens and tissue samples for use in molecular studies. Techniques in Molecular Systematics and Evolution.

[ref-93] Raposo MA, Stopiglia R, Brito GRR, Bockmann FA, Kirwan GM, Gayon J, Dubois A (2017). What really hampers taxonomy and conservation? A riposte to Garnett and Christidis (2017). Zootaxa.

[ref-94] Ratnasingham S, Hebert PDN (2007). BOLD: the barcode of life data system (http://www.barcodinglife.org). Molecular Ecology Notes.

[ref-95] Reiss RA, Schwert DP, Ashworth AC (1995). Field preservation of Coleoptera for molecular genetic analyses. Environmental Entomology.

[ref-96] Richards S, Murali SC (2015). Best practices in insect genome sequencing: what works and what doesn’t. Current Opinion in Insect Science.

[ref-97] Ritter CD, Häggqvist S, Karlsson D, Sääksjärvi I, Muasya AM, Nilsson RH, Antonelli A (2019). Biodiversity assessments in the 21st century: the potential of insect traps to complement environmental samples for estimating eukaryotic and prokaryotic diversity using high-throughput DNA metabarcoding. Genome.

[ref-98] Ritter CD, McCrate G, Nilsson RH, Fearnside PM, Palme U, Antonelli A (2017). Environmental impact assessment in Brazilian Amazonia: challenges and prospects to assess biodiversity. Biological Conservation.

[ref-99] Rochette NC, Catchen JM (2017). Deriving genotypes from RAD-seq short-read data using Stacks. Nature Protocols.

[ref-100] Ruane S, Austin CC (2017). Phylogenomics using formalin-fixed and 100+ year-old intractable natural history specimens. Molecular Ecology Resources.

[ref-101] Ryan SF, Deines JM, Scriber JM, Pfrender ME, Jones SE, Emrich SJ, Hellmann JJ (2018). Climate-mediated hybrid zone movement revealed with genomics, museum collection, and simulation modeling. Proceedings of the National Academy of Sciences of the United States of America.

[ref-102] Sadd BM, Barribeau SM, Bloch G, De Graaf DC, Dearden P, Elsik CG, Gadau J, Grimmelikhuijzen CJP, Hasselmann M, Lozier JD, Robertson HM, Smagghe G, Stolle E, Van Vaerenbergh M, Waterhouse RM, Bornberg-Bauer E, Klasberg S, Bennett AK, Câmara F, Guigó R, Hoff K, Mariotti M, Munoz-Torres M, Murphy T, Santesmasses D, Amdam GV, Beckers M, Beye M, Biewer M, Bitondi MMG, Blaxter ML, Bourke AFG, Brown MJF, Buechel SD, Cameron R, Cappelle K, Carolan JC, Christiaens O, Ciborowski KL, Clarke DF, Colgan TJ, Collins DH, Cridge AG, Dalmay T, Dreier S, Du Plessis L, Duncan E, Erler S, Evans J, Falcon T, Flores K, Freitas FCP, Fuchikawa T, Gempe T, Hartfelder K, Hauser F, Helbing S, Humann FC, Irvine F, Jermiin LS, Johnson CE, Johnson RM, Jones AK, Kadowaki T, Kidner JH, Koch V, Köhler A, Kraus FB, Lattorff HMG, Leask M, Lockett GA, Mallon EB, Antonio DSM, Marxer M, Meeus I, Moritz RFA, Nair A, Näpflin K, Nissen I, Niu J, Nunes FMF, Oakeshott JG, Osborne A, Otte M, Pinheiro DG, Rossié N, Rueppell O, Santos CG, Schmid-Hempel R, Schmitt BD, Schulte C, Simões ZLP, Soares MPM, Swevers L, Winnebeck EC, Wolschin F, Yu N, Zdobnov EM, Aqrawi PK, Blankenburg KP, Coyle M, Francisco L, Hernandez AG, Holder M, Hudson ME, Jackson LR, Jayaseelan J, Joshi V, Kovar C, Lee SL, Mata R, Mathew T, Newsham IF, Ngo R, Okwuonu G, Pham C, Pu LL, Saada N, Santibanez J, Simmons DN, Thornton R, Venkat A, Walden KKO, Wu YQ, Debyser G, Devreese B, Asher C, Blommaert J, Chipman AD, Chittka L, Fouks B, Liu J, O’Neill MP, Sumner S, Puiu D, Qu J, Salzberg SL, Scherer SE, Muzny DM, Richards S, Robinson GE, Gibbs RA, Schmid-Hempel P, Worley KC (2015). The genomes of two key bumblebee species with primitive eusocial organization. Genome Biology.

[ref-103] Sawyer S, Krause J, Guschanski K, Savolainen V, Pääbo S (2012). Temporal patterns of nucleotide misincorporations and DNA fragmentation in ancient DNA. PLOS ONE.

[ref-104] Scheffers BR, Joppa LN, Pimm SL, Laurance WF (2012). What we know and don’t know about Earth’s missing biodiversity. Trends in Ecology & Evolution.

[ref-105] Schloss PD, Westcott SL, Ryabin T, Hall JR, Hartmann M, Hollister EB, Lesniewski RA, Oakley BB, Parks DH, Robinson CJ, Sahl JW, Stres B, Thallinger GG, Van Horn DJ, Weber CF (2009). Introducing mothur: open-source, platform-independent, community-supported software for describing and comparing microbial communities. Applied and Environmental Microbiology.

[ref-106] Schoonvaere K, De Smet L, Smagghe G, Vierstraete A, Braeckman BP, De Graaf DC (2016). Unbiased RNA shotgun metagenomics in social and solitary wild bees detects associations with eukaryote parasites and new viruses. PLOS ONE.

[ref-107] Shendure J, Balasubramanian S, Church GM, Gilbert W, Rogers J, Schloss JA, Waterston RH (2017). DNA sequencing at 40: past, present and future. Nature.

[ref-108] Shi W, Xie S, Chen X, Sun S, Zhou X, Liu L, Gao P, Kyrpides NC, No EG, Yuan JS (2013). Comparative genomic analysis of the endosymbionts of herbivorous insects reveals eco-environmental adaptations: biotechnology applications. PLOS Genetics.

[ref-109] Shokralla S, Gibson JF, King I, Baird DJ, Janzen DH, Hallwachs W, Hajibabaei M (2016). Environmental DNA barcode sequence capture: targeted, PCR-free sequence capture for biodiversity analysis from bulk environmental samples. biorxiv preprint.

[ref-110] Shokralla S, Spall JL, Gibson JF, Hajibabaei M (2012). Next-generation sequencing technologies for environmental DNA research. Molecular Ecology.

[ref-111] Short AEZ, Dikow T, Moreau CS (2018). Entomological collections in the age of big data. Annual Review of Entomology.

[ref-112] Srivathsan A, Ang A, Vogler AP, Meier R (2016). Fecal metagenomics for the simultaneous assessment of diet, parasites, and population genetics of an understudied primate. Frontiers in Zoology.

[ref-113] Srivathsan A, Baloğlu B, Wang W, Tan WX, Bertrand D, Ng AHQ, Boey EJH, Koh JJY, Nagarajan N, Meier R (2018). A MinION^™^-based pipeline for fast and cost-effective DNA barcoding. Molecular Ecology Resources.

[ref-114] Staats M, Erkens RHJ, Van De Vossenberg B, Wieringa JJ, Kraaijeveld K, Stielow B, Geml J, Richardson JE, Bakker FT (2013). Genomic treasure troves: complete genome sequencing of herbarium and insect museum specimens. PLOS ONE.

[ref-115] Stork NE (2018). How many species of insects and other terrestrial arthropods are there on Earth?. Annual Review of Entomology.

[ref-116] Stork NE, McBroom J, Gely C, Hamilton AJ (2015). New approaches narrow global species estimates for beetles, insects, and terrestrial arthropods. Proceedings of the National Academy of Sciences of the United States of America.

[ref-117] Suarez AV, Tsutsui ND (2004). The value of museum collections for research and society. BioScience.

[ref-118] Suchan T, Pitteloud C, Gerasimova NS, Kostikova A, Schmid S, Arrigo N, Pajkovic M, Ronikier M, Alvarez N (2016). Hybridization capture using RAD probes (hyRAD), a new tool for performing genomic analyses on collection specimens. PLOS ONE.

[ref-119] Suen G, Scott JJ, Aylward FO, Adams SM, Tringe SG, Pinto-Tomás AA, Foster CE, Pauly M, Weimer PJ, Barry KW, Goodwin LA, Bouffard P, Li L, Osterberger J, Harkins TT, Slater SC, Donohue TJ, Currie CR (2010). An insect herbivore microbiome with high plant biomass-degrading capacity. PLOS Genetics.

[ref-120] Suyama Y, Matsuki Y (2015). MIG-seq: an effective PCR-based method for genome-wide single-nucleotide polymorphism genotyping using the next-generation sequencing platform. Scientific Reports.

[ref-121] Taberlet P, Coissac E, Pompanon F, Brochmann C, Willerslev E (2012). Towards next-generation biodiversity assessment using DNA metabarcoding. Molecular Ecology.

[ref-122] Tagliavia M, Massa B, Albanese I, La Farina M (2011). DNA extraction from Orthoptera museum specimens. Analytical Letters.

[ref-123] Tang M, Tan M, Meng G, Yang S, Su X, Liu S, Song W, Li Y, Wu Q, Zhang A, Zhou X (2014). Multiplex sequencing of pooled mitochondrial genomes—a crucial step toward biodiversity analysis using mito-metagenomics. Nucleic Acids Research.

[ref-124] Tedersoo L, Anslan S, Bahram M, Põlme S, Riit T, Liiv I, Kõljalg U, Kisand V, Nilsson RH, Hildebrand F, Bork P, Abarenkov K (2015). Shotgun metagenomes and multiple primer pair-barcode combinations of amplicons reveal biases in metabarcoding analyses of fungi. MycoKeys.

[ref-125] Thompson JF, Milos PM (2011). The properties and applications of single-molecule DNA sequencing. Genome Biology.

[ref-126] Thomsen PF, Elias S, Gilbert MTP, Haile J, Munch K, Kuzmina S, Froese DG, Sher A, Holdaway RN, Willerslev E (2009). Non-destructive sampling of ancient insect DNA. PLOS ONE.

[ref-127] Thorpe SE (2017). Is photography-based taxonomy really inadequate, unnecessary, and potentially harmful for biological sciences? A reply to Ceríaco et al. (2016). Zootaxa.

[ref-128] Timmermans MJTN, Viberg C, Martin G, Hopkins K, Vogler AP (2016). Rapid assembly of taxonomically validated mitochondrial genomes from historical insect collections. Biological Journal of the Linnean Society.

[ref-129] Tin MM-Y, Economo EP, Mikheyev AS (2014). Sequencing degraded DNA from non-destructively sampled museum specimens for RAD-tagging and low-coverage shotgun phylogenetics. PLOS ONE.

[ref-130] Toju H (2015). High-throughput DNA barcoding for ecological network studies. Population Ecology.

[ref-131] Veijalainen A, Wahlberg N, Broad GR, Erwin TL, Longino JT, Sääksjärvi IE (2012). Unprecedented ichneumonid parasitoid wasp diversity in tropical forests. Proceedings of the Royal Society B: Biological Sciences.

[ref-132] Vellend M (2017). The biodiversity conservation paradox. American Scientist.

[ref-133] Vesterinen EJ, Ruokolainen L, Wahlberg N, Peña C, Roslin T, Laine VN, Vasko V, Sääksjärvi IE, Norrdahl K, Lilley TM (2016). What you need is what you eat? Prey selection by the bat *Myotis daubentonii*. Molecular Ecology.

[ref-134] Vogel G (2017). Where have all the insects gone?. Science.

[ref-135] Wachi N, Matsubayashi KW, Maeto K (2018). Application of next-generation sequencing to the study of non-model insects. Entomological Science.

[ref-136] Wheeler QD, Raven PH, Wilson EO (2004). Taxonomy: impediment or expedient?. Science.

[ref-137] Yeates DK, Meusemann K, Trautwein M, Wiegmann B, Zwick A (2016). Power, resolution and bias: recent advances in insect phylogeny driven by the genomic revolution. Current Opinion in Insect Science.

[ref-138] Yin C, Shen G, Guo D, Wang S, Ma X, Xiao H, Liu J, Zhang Z, Liu Y, Zhang Y, Yu K, Huang S, Li F (2016). InsectBase: a resource for insect genomes and transcriptomes. Nucleic Acids Research.

[ref-139] Young AD, Lemmon AR, Skevington JH, Mengual X, Ståhls G, Reemer M, Jordaens K, Kelso S, Lemmon EM, Hauser M, De Meyer M, Misof B, Wiegmann BM (2016). Anchored enrichment dataset for true flies (order Diptera) reveals insights into the phylogeny of flower flies (family Syrphidae). BMC Evolutionary Biology.

[ref-140] Zhou X, Li Y, Liu S, Yang Q, Su X, Zhou L, Tang M, Fu R, Li J, Huang Q (2013). Ultra-deep sequencing enables high-fidelity recovery of biodiversity for bulk arthropod samples without PCR amplification. GigaScience.

